# Effects of Apple Form on Energy Intake During a Mid-Afternoon Snack: A Preload Paradigm Study in School-Aged Children

**DOI:** 10.3389/fnut.2021.620335

**Published:** 2021-03-26

**Authors:** Camille Schwartz, Ophélie Person, Emilie Szleper, Sophie Nicklaus, Carole Tournier

**Affiliations:** Centre des Sciences du Goût et de l'Alimentation, AgroSup Dijon, CNRS, INRAE, Université Bourgogne Franche-Comté, Dijon, France

**Keywords:** food texture, preload paradigm, Food Oral Processing, satiation, CEBQ, eating rate, eating behavior, mastication

## Abstract

Consuming foods with a form or a texture that requires longer oral processing is a way to decrease food intake. Although this approach is promising for leveraging healthier eating patterns in adults, it has never been explored in children. This study evaluated whether starting a mid-afternoon snack by eating either apple segments or applesauce would modify hunger and subsequent food intake during this meal. Forty-four children (8–10 years old) participated in two videotaped mid-afternoon snacks, during which they received one of the two forms of apple as a food preload followed 10 min later by *ad libitum* consumption of sweetened cottage cheese. They self-reported their level of hunger throughout consumption, and the weight of cottage cheese consumed was determined at the end of the snack. Children's chewing capabilities and eating traits were parent-reported. Eating a raw apple increased oral exposure time and decreased bite size compared to eating applesauce. However, neither the reported hunger nor consecutive food intake were modified. Regardless of the meal, children eating fast had a higher *ad libitum* energy intake. The individual eating rate for the cottage cheese was correlated with the eating rate observed for applesauce but not for apple segments, the latter being associated with children's chewing difficulties. This study suggests that the form of a fruit offered at the start of a mid-afternoon snack does not impact food intake; the findings clearly call for more exploration of satiation mechanisms related to food texture properties among children and indicate the need to consider children's oral processing skills.

## Introduction

Childhood obesity is a public health concern in all regions of the world: “over 340 million children and adolescents aged 5–19 years old were overweight or obese in 2016” (1). Researchers, governmental policy makers, and food industries have the responsibility to think about new strategies to leverage healthier eating behaviors and decrease energy intake and, in turn, weight gain in children.

In adults, there is strong evidence that food oral processing should be considered as a determinant of food intake. Manipulations of food texture in such a way that decreases bite size and/or increases chewing per bite (and thus oral exposure time) yield a decrease in intake of these foods (2). It has been suggested that a 20% reduction in eating rate (g/min) is necessary to reach a 10–15% energy intake reduction (3). Thus, a large set of studies investigated the efficiency of changing food form, textural properties, textural complexity, or shape in reducing intake [see many reviews for details: (2, 4–10)]. Large effects are usually observed when the food form is modified (i.e., from liquid to semisolid) (2). Interestingly, changing the food form has also been found to impact the intake of other foods eaten during the same meal (and yet a meal is often a combination of several dishes). In their study, Flood-Obbagy and Rolls (11) used a preload paradigm and tested the effect of starting a meal by consuming apple offered as juice (liquid), juice with added fiber (liquid), applesauce (semisolid), and apple segments (solid) (all forms being matched for energy content, weight, energy density, and ingestion rate) on subsequent *ad libitum* energy intake at the same meal. They observed that apple segments reduced the subsequent food intake more than applesauce or apple juice. This suggests that offering fruit (which is advocated in children) with a different form (raw rather than pureed or as a juice) might be a way to decrease the consumption of other foods offered within the same meal and, in turn, to reduce energy intake. However, although research on the impact of manipulation of food oral processing (though a manipulation of the food texture or the food form) on intake has been widely investigated in adults, very few studies have explored this in children. These studies suggest that the shape and serving size of a vegetable (whole vs. diced carrots) play a role in *ad libitum* intake (12, 13). More research is warranted with child-tailored paradigms, especially on the potential of food manipulations inducing changes in food oral processing for preventing overconsumption during a meal.

Food liking and familiarity are strong drivers of food intake in children (14). Changes in food texture affect acceptance in 3- to 4-year-old children (15) and preference in school-aged children (16). In addition to food liking, food familiarity, and texture acceptance, individual oral processing skills and behavior during a meal are other individual determinants of food intake, particularly through the induced eating rate. Among 4.5-year-old children enrolled in the GUSTO Singaporean cohort, children described as “fast eaters” had a larger average bite size, fewer chews per gram of food, and higher energy intake than children who were “slow eaters” (17, 18). Interestingly, overweight children were more likely to be characterized as “fast eaters” (17). In addition, another study showed that the number of mouthfuls of food/minute observed at 4 years predicted changes in body mass index (BMI) from 4 to 6 years, suggesting that a rapid eating style may be a behavioral marker for the development of childhood obesity (19). Certainly, the development of masticatory efficiency and changes in oral physiology occurring during childhood play a role in the individual eating rate and the ability to cope with more or less complex textured foods. Indeed, between 6 and 10 years, changes in jaw displacements, dentition stage (early mixed dentition), and development of bite force influence the masticatory behavior of children (20). Some children might encounter chewing difficulties when eating harder foods, for example. Pioneering work from Linas and colleagues (21, 22) reported that children presenting early childhood caries (ECC) have more eating difficulties and produce a reduced breakdown of hard foods in the oral cavity (as assessed from the granulometry of their food bolus collected at swallowing). Less information is available about the variability in oral processing behaviors among children with healthy dentition and how this can influence eating rate during a meal. Finally, children's eating rate is associated with their parental reports of food fussiness, food enjoyment, and satiety responsiveness [as evaluated by the Children's Eating Behavior Questionnaire (CEBQ)] (23), suggesting the need to also consider children's appetitive traits when studying their oral processing behavior.

Within this context, our main objective was to probe whether changing the form (semisolid to solid) of a fruit offered at the beginning of a mid-afternoon snack [“goûter,” which is a very common practice among children in France (24)] would influence the level of reported hunger and subsequent intake of another food offered within the same mid-afternoon snack (refer here after as snack or meal) in children. A secondary objective was to explore the variability in children's oral processing behaviors when eating the different foods offered during the snack and their potential links with parent reports of children's chewing behavior, chewing difficulties, and appetitive traits. Our hypotheses were as follows: (i) consuming a raw apple preload would increase oral exposure time and subsequently reduce *ad libitum* intake during the snack compared to a pureed apple preload; (ii) high *ad libitum* intake would be related to a high eating rate (i.e., fast eaters are eating more); and (iii) at an individual level, eating rate would be associated with appetite traits and chewing behavior for chewable food.

## Methods

### Participants

Children aged between 8 and 10 years old were recruited in 2019 through flyers distributed in schools and afterschool leisure centers and by contacting parents registered in the ChemoSens Platform's PanelSens database [Commission Nationale de l'Informatique et des Libertés (CNIL, n 1148039)] to participate in two mid-afternoon snacks in the laboratory. To be included, children had to attend primary schools (3rd, 4th, and 5th grades), regularly consume a mid-afternoon snack, and be consumers of apple and cottage cheese (“fromage blanc” in French). Exclusion criteria were diagnosed feeding disorder, diabetes, and/or food allergy. The methodology complies with the Declaration of Helsinki and was approved by an ethics committee (Comité de Protection des Personnes Ile-de-France II IDRCB 2019-A00890-57). Written informed consent was obtained from parents prior to the start of the study as well as children's oral assent. Our objective was to recruit a sample of 50 children. In the absence of a similar study in children, we were not able to run a power analysis, so we based our sample size choice on other studies conducted in children [e.g., a study with 40 school-aged children highlighted a significant link between a decrease in liking (for a fruit puree) and a decrease in the level of hunger during mid-afternoon snacks (25)] or in adults (11).

### Food Products Offered During the Snack

**Figure 1:**
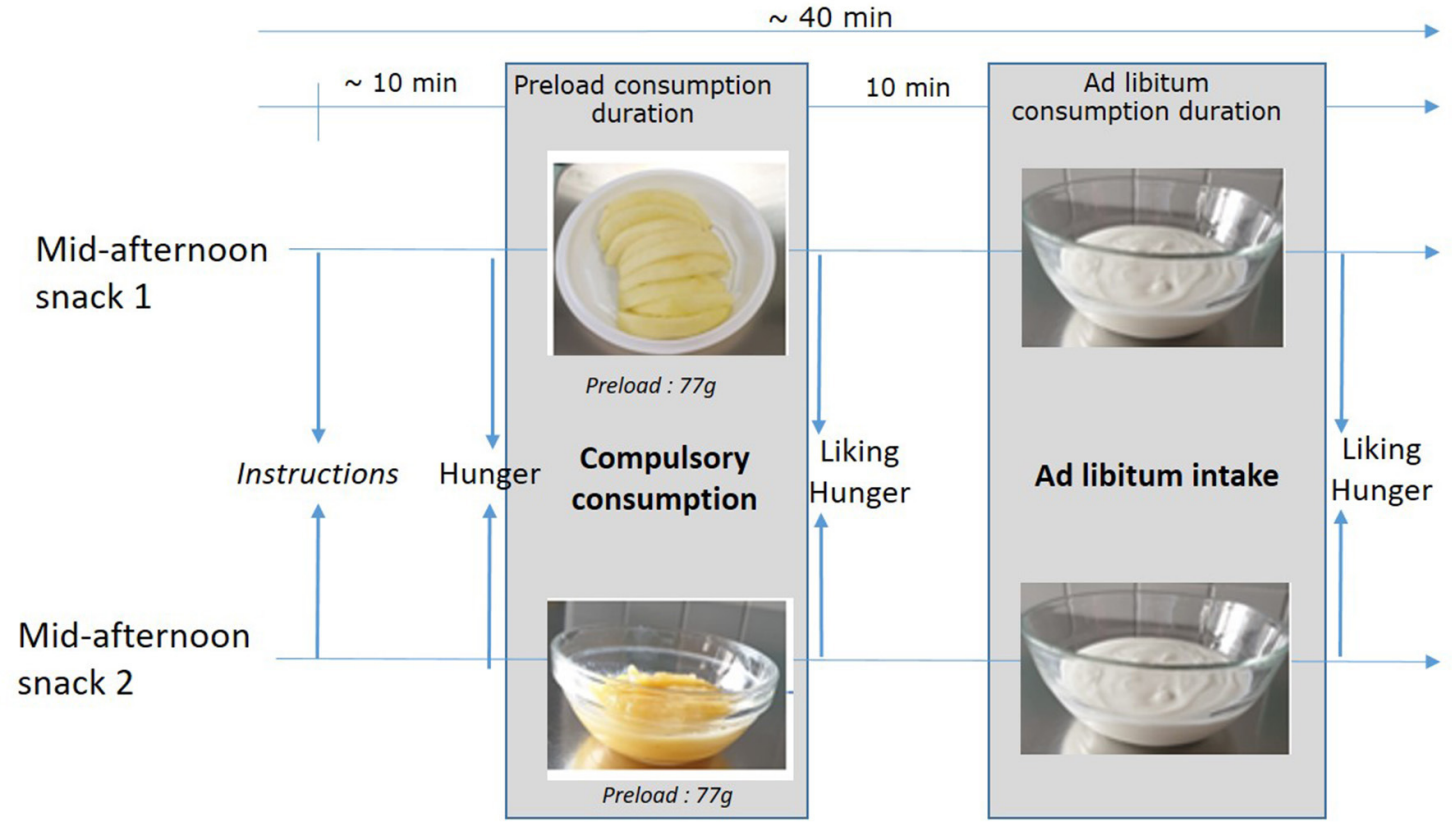
Mid-afternoon snack design (preload and *ad libitum* consumption durations were left to the discretion of the child and are presented in Table 4; mid-afternoon snacks starting with apple segments or applesauce were balanced between children).

Two foods regularly consumed by children during mid-afternoon snacks were chosen: apple and sweetened cottage cheese (24, 26). Based on the study by Flood-Obbagy and Rolls (11), two forms of apple were chosen and were matched for energy density (ED) and weight: raw apple segments and applesauce (see Figure 1). The applesauce for the whole study was prepared once in the laboratory according to the HACCP regulation for hygiene and safety and was frozen at −18°C and then batch-thawed for each experimental day. Pink Lady® apples were peeled, seeded, cooked, and mixed using a Vorwerk robot (Thermomix®); water was added to compensate for water losses during the process to ensure that apple segments and applesauce were isocaloric (a dry extract analysis showed that the applesauce contained 84.8 ± 0.1% water, and the apple segments contained 83.9 ± 0.2% water). The ED of apple segment Pink Lady® was 54 kcal/100 g. Sweetened (5% *w*/*w*) cottage cheese (Jockey®, Danone; per 100 g: 3 g of fat, 4.4 g of carbohydrate, and 6.9 g of protein) was chosen for the subsequent *ad libitum* meal (Figure 1). The ED of the cottage cheese (3.3% of fat, Jockey®, DANONE) was 76 kcal/100 g. This choice was similar to the study by Bouhlal and colleagues (27, 28) in 2- to 3-year-old children, which showed that this food was liked but not too much and would probably then limit overconsumption.

### Study Timeline and Measurements

Children participated in two mid-afternoon snacks (at 5:10 pm or 6:10 pm) in the laboratory with a 4–14 days delay in between; each mid-afternoon snack lasted approximately 40 min. Having a mid-afternoon snack is culturally very common for children in France (24), is usually composed of several foods (among which fruit purees are very common with the advent of fruit purees in soft pouches), and represents 17% of daily energy intake in 0–10 year olds (26). The timing was chosen so that children could come to the laboratory after school. This is an average of 2.5 h before the usual dinner time observed in France (29).

We chose a preload paradigm, as it is a design allowing direct observation of intake in a well-controlled environment (30). It consists of a randomized crossover satiation trial with participants eating the preload and then eating a test food *ad libitum*. The delay between the start of the preload and the start of the test food is usually no less than 5 min (30). It was theorized that “up to 15 min after intake the delay is consistent with a meal” and thus reflects satiation effect during the same meal (31). Using this design enables us to directly measure the impact of the preload properties (modification of the food form) on satiation and the consumption of the other food served during the same eating occasion while controlling for potential effects linked to texture acceptance given that the consumption of the food preload is compulsory and that the two preloads are matched for weight. With another possible design, “concurrent evaluation,” distinguishing the effect of food texture acceptance from the effect of the food form on consumption is more difficult. The study design is presented in Figure 1.

A fixed preload quantity was offered, and the consumption of this entire portion was compulsory, but the eating duration was left to the discretion of the child. Then, after a 10-min delay, cottage cheese was served *ad libitum*. The presentation of the food preload was balanced (i.e., half of the children started the first mid-afternoon snack with apple segments, whereas the other half started with applesauce). The chosen portion was 77 g for the preload [apple segments were served into 10 segments of 7 g; this is equivalent to 16% of usual energy intake from mid-afternoon snacks in 0- to 10-year-old children, as reported by the French INCA3 study (26)]. Portion sizes were defined from piloting tests and corresponded to almost the weight of an apple puree pouch (90 g in general), which would be a relevant portion of what is offered during a mid-afternoon snack meal, in particular with other offered foods. The portion of cottage cheese was 500 g: 400 g was served first in a bowl with a spoon; then, each child could ask for two supplemental portions of 50 g to ensure *ad libitum* consumption (500 g of sweetened cottage cheese is equivalent to 181% of usual energy intake from mid-afternoon snacks). Both meals were organized in a sensory evaluation room; parents were waiting for their children in a room next door. Instructions were given to a group of five–six children collectively by an experimenter at the beginning of the meal, and then children were isolated in individual booths to reduce distraction.

### Evaluation of Hunger, Liking, and *ad libitum* Intake

The timeline of the snack was as follows (Figure 1): first, children evaluated their initial hunger through a dedicated child-tailored 10-cm visual analog scale (VAS) after being collectively trained to use it. It consists of a visual representation (i.e., cartoon figures, one scale for girls and one for boys) of five levels of hunger varying from “I am not hungry at all. My stomach is completely full, and I cannot eat anymore” to “I am very hungry! My stomach is truly really empty and is rumbling at lot” [see (25, 32) for details]. Then, they ate the apple preload entirely and rated their liking on a dedicated child-tailored 10-cm VAS punctuated by smileys [see (25, 32)] and evaluated again their level of hunger (Figure 1). After this, and for 10 min that were strictly controlled, they played an online game based on the principle of “where's Wally?”. By the end of this delay, children were offered cottage cheese. They were free to eat as much as they wanted to until they were no longer hungry. Once they stopped eating, they evaluated their liking for the cottage cheese and their level of hunger. Eaten quantities for all foods (preloads and *ad libitum* foods) were evaluated by weighing the containers before and after consumption (1 g, Soehnle Page, Benfeld, Germany).

### Coding of Oral Processing Behaviors

In each booth, children were videotaped to make the description of their oral processing behaviors possible and assess the consumption duration for both preload types. Behaviors were coded using Noldus The Observer® software by a single trained video coder. The coding scheme (see Table 1) was inspired by the one used in Fogel and colleagues (17). The validity of the coding scheme was checked by estimating the agreement with another trained coder (Pearson correlation coefficients for the coded variables: *r* = 0.75 to 0.99) on a subsample of 10 randomly selected videos.

**Table 1:** Video-coded oral processing behaviors, methodological parameters, and output variables.

	**Definition of behaviors**	Coding scheme^*^	**Output variables**
1. Consumption duration (segments and applesauce)	Ingestion behavior between the start of the consumption corresponding to the first lip–food contact, when the child closed his mouth after the first bite until the end of the consumption corresponding to the swallowing of the last bite.	State event	Total consumption duration throughout the consumption episode (s): **Duration (s)** The average eating rate was calculated by dividing the grams consumed over the consumption duration: **EatingRate (g/min)**
2. Bite (segments and applesauce) number and duration	Duration from the time when the food enters into the mouth up to the swallows (sequence of biting/eaten mouthfuls and swallows)—this duration does not include breaks (when no food was into the mouth)	State and point event	Number of bites/mouthfuls to eat the entire portion: **Bites (*****N*****)** Cumulated duration of bites/mouthfuls to eat the entire portion (consumption duration–cumulative duration of breaks): **OralExposureTime (s)** The average bite size was calculated by dividing the grams consumed by the number of bites/mouthfuls to eat the entire portion: **BiteSize (g per bite/mouthful)**
3. Chew or masticatory cycle (segments only)	Up and down movements of the jaws during apple segments consumption. This behavior was coded for 3 out of 10 segments^a^	Point event	Number of up and down movements of the jaws for three apple segments: **MasticatoryCycles (*****N*****)**

* *State events have a duration (e.g., consumption duration), point events do not (e.g., number of chews)*.

a *For a majority of children, this was done for segments 4, 5, and 6; when necessary due to problems of visibility on the videos, this was done for segments 3, 4, and 5 or 5, 6, and 7*.

### Parental Self-Report of Children's Chewing Behavior and Eating Temperament

Parents were asked to fill out questionnaires to evaluate some facets of their child's eating behavior. Their perception of their child's chewing difficulties and chewing behavior traits was assessed via five items (see Table 2). To define the items, we were inspired by previous works (33, 34) but adapted the items for this study to assess only children's difficulties coping with hard/difficult textures and children's chewing behavior and with a limited number of items. Parents answered each item with a three-category answer for each item (“yes,” “no,” and “I don't know”). Answers “yes” were coded as 1, “no” as 2, and “I do not know” as missing. Two scores were derived (as the mean for the concerned items): one relates to the difficulty of coping with hard/difficult textures (P-noDiff_HardTexture; the higher this score is, the lower the difficulty of coping with hard/difficult textures) and the other describes the extent to which children chew their food before swallowing (P-Chewing; the higher the score, the better the child chews the food before swallowing). The Cronbach's α values were satisfactory for both dimensions [P-noDiff_HardTexture (2 items) = 0.70; P-Chewing (three items) = 0.89].

**Table 2:** Parental questionnaire to evaluate their child's chewing difficulty and chewing behavior traits.

	**Oui/*Yes*** ***Coded as 1***	**Non/*No*** ***Coded as 2***	**Je ne sais pas/** ***I do not know***
**1.R (B)** Mon enfant prend le temps de bien mâcher ses aliments*My child takes the time to chew his food*	□	□	□
**2. (A)** Mon enfant refuse de manger certains aliments parce qu'il les trouve trop difficiles à mâcher*My child refuses to eat certain foods because he/she finds them too difficult to chew*	□ Si oui, lesquels: *If yes, which ones*	□	□
**3. (B)** Lorsqu'il/elle mange, mon enfant a tendance à mettre trop de nourriture/à prendre de trop grosses bouchées, par rapport à la taille de sa bouche*When he/she eats, my child tends to put too much food on his/her fork/spoon or takes too large mouthfuls in relation to the size of his/her mouth*	□	□	□
**4. (B)** Mon enfant mâche très peu sa nourriture, j'ai l'impression qu'il/elle avale ‘tout rond'*My child doesn't chew his/her food much, I have the impression that he/she eats everything whole*	□	□	□
**8. (A)** J'ai besoin de couper la viande de mon enfant en très petits morceaux pour l'aider à manger*I need to cut my child's meat into very small pieces to help him/her to eat*	□	□	□

*Two composite scores were extracted: one relates to the difficulty of coping with hard/difficult textures (P-noDiff_HardTexture; (A) items in the table), whereas the other relates to the fact that the child eats without chewing (P-Chewing; (B) items in the table; R indicates that the item is reversed)*.

Children's eating temperaments were assessed with the Child Eating Behavior Questionnaire [CEBQ (35)], and some dimensions only were retained for this analysis: slowness in eating (SE) (e.g., “My child eats slowly”), satiety responsiveness (SR) (e.g., “My child leaves food on his/her plate at the end of a meal” or “My child gets full before his/her meal is finished”), enjoyment of food (EF) (e.g., “My child loves food”), food responsiveness (FR) (for example, “My child is always asking for food”), and food fussiness (FF) (e.g., “My child is difficult to please with meals”), as done by Fogel and colleagues (23). Parents had to indicate how much this was true for their child (from 1: never to 5: always). The score for each dimension is the mean of the different items, and by definition, it ranges from 1 to 5. The Cronbach's α values were satisfactory for the five dimensions (SR = 0.80; SE = 0.79; EF = 0.74; FR = 0.86; FF = 0.90).

### Anthropometric Measurements

Children's weight and height were measured in duplicate by a trained experimenter. Children were weighed to the nearest 0.1 kg using a digital scale (PHARO 200, Soehnle, Benfeld, Germany) without shoes. Their length was measured to the nearest 0.1 cm using a stadiometer (TANITA Leicester, Birmingham, UK). Weight and height were transformed into BMI *z*-scores (BMIz) corrected for age and sex according to the WHO child growth reference for school-aged children and adolescents (36).

### Statistical Analysis

SAS 9.3 for Windows (SAS Institute Inc., Cary, NC) was used to perform the analyses. The results are expressed as the means ± SDs. Significance was set at *p* < 0.05. To check that the food oral processing behaviors and oral exposure time thereof were significantly different between the two types of preload, paired Student's *t*-tests were calculated on the oral processing variables. Differences in liking (0–10) between both preloads and cottage cheese after each preload type were also studied (paired *t*-tests).

#### Impact of Apple Preload Form on Subsequent Hunger and *ad libitum* Intake

Despite the compulsory consumption of the apple preload, some children could not manage to eat it all. A minimal preload consumption of 80% (i.e., at least 62 g of 77 g) was set to analyze the cottage cheese intake data (37). We verified that preload intake was not different between preload forms using an analysis of variance (subject, preload). A mixed model was calculated to evaluate whether the hunger level changed over the session: the dependent variable was the hunger level (0–10), with “subject” as a random factor and with time (three levels: before preload, after preload, and after subsequent *ad libitum* consumption), preload type (two levels: apple segments and applesauce), and the preload type × time interaction as the fixed effects. The mean percentage of hunger decrease due to preload consumption [i.e., (rating score before preload consumption minus rating score after consumption) divided by rating score before preload consumption] and due to cottage cheese consumption (similarly, difference between after preload consumption and after *ad libitum* consumption) were determined and analyzed using ANOVA (subject, preload).

The impact of preload type on cottage cheese *ad libitum* intake (in grams or in kilocalories) was assessed using a mixed model adjusted for initial level of hunger, child age, BMIz, and sex. The correlation between cottage cheese intake and the observed eating rate was assessed using the Kendall correlation coefficient (*p* < 0.05).

#### Eating Rates: Impact of Food Form and Links With Parental Reports of Chewing Behavior and Eating Temperament

Kendall correlation coefficients were calculated to assess the associations between eating rates observed for the cottage cheese and for the preload eaten during the same snack. The effect of chewing difficulties (P-NoDiff_HardTexture) and chewing activity before swallowing (P-Chewing) on eating rate was assessed using one-way analysis of variance with chewing level as a factor with Student Newman–Keuls *post hoc* analysis. P-NoDiff_HardTexture was obtained by means of two items and therefore has three levels: 1 (the child has difficulties), 1.5 (the child sometimes has difficulties), or 2 (the child has no difficulties). When a significant effect was observed on eating rate, we further explored the effect of chewing difficulties on observed oral processing behaviors (bite size, number of bites, oral exposure time, and number of masticatory cycles) with a Bonferroni adjustment (alpha level of 0.013 = 0.05/4 per test). Associations between eating rate and CEBQ dimensions (SR, SE, EF, FR, and FF) were explored using Kendall correlation coefficients and Bonferroni adjustment (alpha level of 0.01 = 0.05/5 per test) to correct for multiple testing.

## Results

### Participants

Fifty children agreed to participate, but two withdrew before coming to the sessions, and four children did not meet the minimal preload consumption (three children ate less than 80% of the apple segments, and one child ate less than 80% of both apple preloads). Data for 44 children were available (23 males). Twenty-four children were followed by a dentist, and six had ongoing orthodontic treatment. They were on average 9.1 ± 0.9 years old and had, on average, a healthy weight status (Table 3).

**Table 3:** Characteristics of the participating children (*N* = 44).

	**Mean**	**SD**	**Min**	**Max**
Height (cm)	139.5	9.0	126.1	167.6
Weight (kg)	33.2	7.1	23.8	54.5
BMI-for-age *z*-score	0.28	1.01	−2.17	2.42

The preloads were equally liked (apple segments = 8.1 ± 1.9, applesauce = 7.9 ± 1.6; *F*(1, 87) = 0.3, *p* > 0.05). The cottage cheese was equally liked (*F*(1, 87) = 1.0, *p* > 0.05) after apple segments (8.4 ± 1.7) or applesauce (8.6 ± 1.5).

### Impact of Preload Form on Oral Exposure Time

The hypothesis that the preload form would affect oral processing duration was verified (Table 4): eating 77 g of apple segments required significantly more time than eating the same weight of applesauce [4.2 ± 1.6 min (*N* = 40) vs. 1.1 ± 0.4 min (*N* = 42), respectively; *t* (37) = 12.20, *p* < 0.0001]. The average difference in consumption duration between applesauce and apple segments was 3.1 ± 1.6 min (min = 0.9, max = 9.9 min). This is related to a longer oral exposure time, which reflects effective oral processing time, characterized by more bites, a smaller bite size, and a slower rate of eating (all *p* < 0.05, Table 4).

**Table 4:** Oral processing behavior variables (videotape coding) when eating both preload types and cottage cheese.

	**Apple segments**					**Applesauce**						
	***N***	**Mean**	**SD**	**Min**	**Max**	***N***	**Mean**	**SD**	**Min**	**Max**	*t* (df)^**^	***P***
**Duration (s)**	40	255	97	128	681	42	70	24	25	122	12.2 (37)	<0.0001
**OralExposureTime (s)**	37	227	90	113	629	42	68	24	25	122	10.6 (34)	<0.0001
**Bite (*****N*****)**	37	22	6	12	37	42	15	5	6	27	5.2 (34)	<0.0001
**BiteSize (g/bite)**	37	4	1	2	6	42	6	2	3	13	−5.2 (34)	<0.0001
**EatingRate (g/min)**	40	20	6	7	36	42	74	29	37	183	−14.3 (37)	<0.0001
**MasticatoryCycles (*****N*****)**	26	81	27	45	143							
***Ad lib*** cottage cheese duration^*^ (s)	37	142	65	47	352	40	125	50	24	212	1.8 (33)	0.0860
***Ad lib*** cottage cheese eating rate^*^ (g/min)	37	90	38	29	190	40	92	44	33	233	0.1 (33)	0.9033

* Values for the cottage cheese consumed after each preload (apple segments or applesauce);

** *t(df): Student's t-test*.

### Effect of Preload Form on Hunger and *ad libitum* Intake

**Figure 2:**
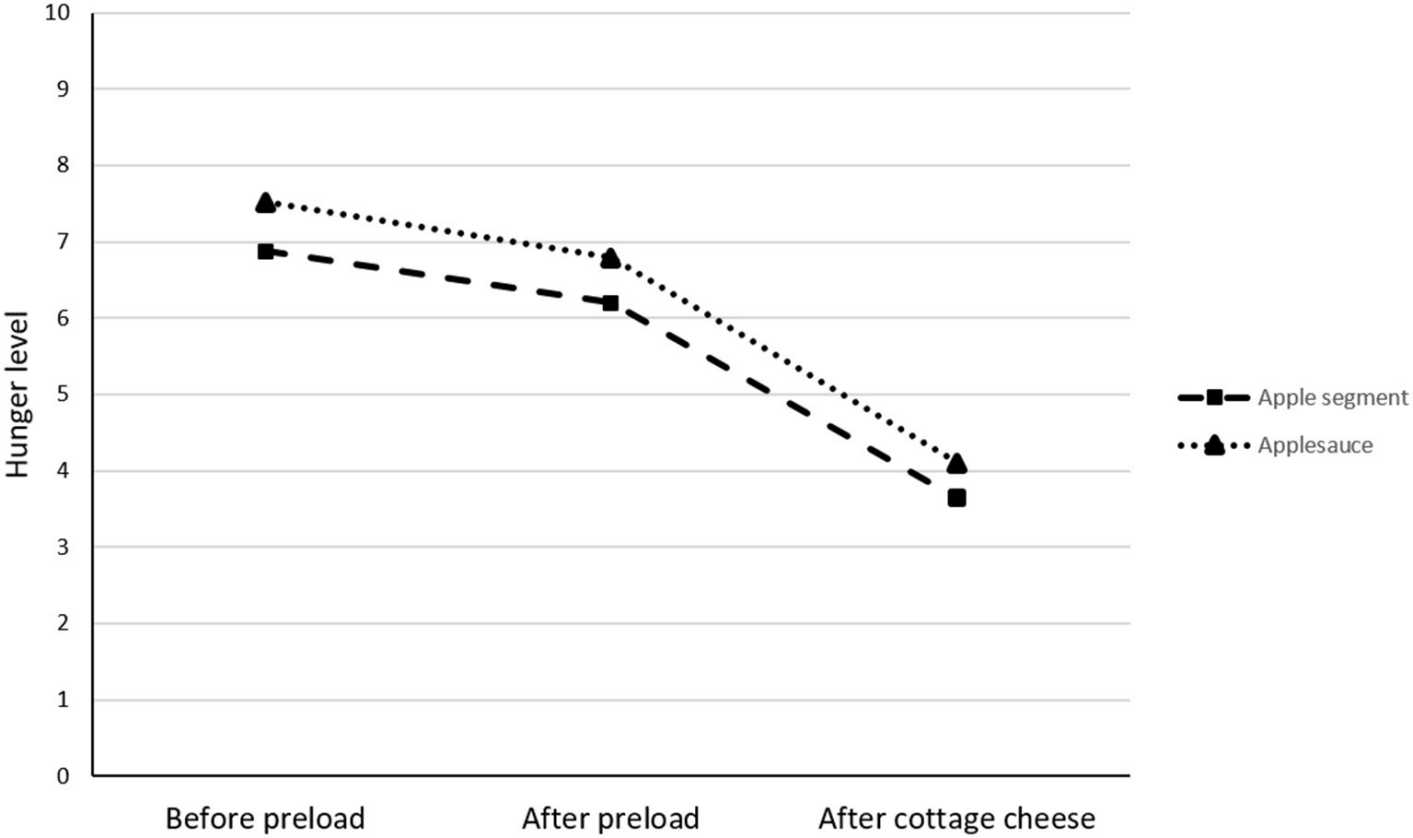
Hunger ratings (mean ± SD) for the 44 children before and after preload and after cottage cheese consumption. The hunger level significantly decreased over the course of the mid-afternoon snack regardless of the preload type consumed (time, *F*(2, 215) = 115.47, *p* < 0.0001; preload type, *F*(1, 215) = 9.07, *p* = 0.0029), but the time × preload type interaction was not significant (*p* = 0.93).

The average preload intake was 76.7 ± 2.1 g for the apple segments and 76.0 ± 0.7 g for the applesauce (*p* > 0.05). Children were quite hungry before preload consumption, and they reported being hungrier at the start of the applesauce snack (7.5 ± 1.9) than at the start of the apple segment snack (6.9 ± 2.0) (*F*(1, 43) = 5.2, *p* < 0.05; Figure 2). The hunger level significantly decreased over the course of the mid-afternoon snack regardless of the preload type consumed [time, *F*(2, 215) = 115.47, *p* < 0.0001; preload type *F*(1, 215) = 9.07, *p* = 0.003, but the time × preload type interaction was not significant (*p* = 0.93)]. Therefore, despite differences in hunger observed at the beginning of the snacks, the hunger decrease due to preload consumption was similar for both the applesauce and apple segment snacks (8.2 and 7.7%, respectively; *p* > 0.05) and was also similar after cottage cheese consumption (39.3 and 40.8% for the applesauce and apple segment snacks, respectively) (*p* > 0.05, Figure 2).

On average, 208 ± 120 g of cottage cheese was consumed after eating the apple segments, whereas 188 ± 107 g was consumed after eating the applesauce. We did not observe any effect of the preload type on *ad libitum* intake of cottage cheese afterwards, either when considering the cottage cheese alone (*p* = 0.20) or the total intake (apple preload + cottage cheese; *p* = 0.19) (Table 5).

**Table 5:** Weight and energy intake of cottage cheese after each preload (mean ± SD).

	**Preload type**	
	**Apple segment**	**Applesauce**
**Cottage cheese intake**		
Weight (g)	208 ± 120	188 ± 107
Energy (kcal)	192 ± 110	173 ± 99
**Total intake (preload + cottage cheese)**		
Weight (g)	285 ± 120	264 ± 107
Energy (kcal)	233 ± 110	214 ± 99

In both sessions, cottage cheese intake was highly correlated with the observed eating rate (τ = 0.51, *p* < 0.0001, *N* = 37 for the session with apple segments and τ = 0.50, *p* < 0.0001, *N* = 40 for the session with applesauce). Thus, children who ate cottage cheese quickly had a higher *ad libitum* intake than those who ate at a slower rate. The eating rate of cottage cheese was consistent between both sessions (*N* = 34, τ = 0.50, *p* < 0.0001).

### Eating Rates: Links With Food Form and Individual Eating Temperament

We explored whether the eating rate observed for cottage cheese was consistent with those observed for the applesauce and apple segments. A significant correlation was observed between the eating rates of cottage cheese and applesauce (*N* = 40; τ = 0.44, *p* < 0.0001) but not between the eating rates of cottage cheese and apple segments (*N* = 37; τ = 0.11, *p* = 0.32).

Individual factors potentially influencing the preload eating rate were studied, including chewing behavior and eating temperament. A significant association between parental reports of children's chewing difficulties (P-noDiff_HardTexture) and the actual children's eating rate was observed in the case of apple segments (*F*(2, 37) = 3.6, *p* = 0.04) but not of applesauce (*p* = 0.09). No associations were observed between eating rate and parental report of children's chewing behavior before swallowing (P-Chewing) or any dimension of the CEBQ.

Further exploration revealed that children with chewing difficulties took significantly more time (*F*(2, 37) = 5.3, *p* = 0.01) to eat the apple segments than children with sometimes or without difficulties [6.0 ± 2.7 min (*n* = 6) vs. 4.1 ± 1.1 (*n* = 7) and 3.9 ± 1.1 min (*n* = 27), respectively]. They required significantly more bites (29.7 ± 6.5 bites vs. 21.9 ± 4.4 and 19.3 ± 4.4 bites; *F*(2, 36) = 11.6, *p* < 0.001) of smaller size (2.7 ± 0.7 g vs. 3.6 ± 0.8 and 4.1 ± 0.9 g, respectively; *F*(2, 36) = 6.6, *p* = 0.004).

## Discussion

This study investigated the impact of fruit form offered at the beginning of a mid-afternoon snack on reported hunger and subsequent *ad libitum* intake of another food offered at the snack. Our results collected in healthy weight, school-aged children under controlled laboratory conditions showed, as expected, that eating apple segments took significantly more time and required more oral processing behaviors than eating the same quantity of applesauce matched for energy density. However, the main hypothesis of this work, based on a previous study in adults, was not confirmed: modifying the preload form (semisolid to solid) did not affect the level of hunger or the subsequent *ad libitum* intake of cottage cheese differently. In other respect, regardless of the preload, the cottage cheese *ad libitum* intake was highly correlated with the eating rate: fast eaters ate more during the snack. Nevertheless, children's eating rate was not consistent across foods: fast eaters of semisolid foods (cottage cheese and applesauce) were not necessarily fast eaters of hard foods, here raw apple segments. Moreover, child chewing difficulties as reported by their parents were negatively associated with the eating rate observed for chewable apple segments.

This study was inspired by a previous study in adults (11) and was adapted to children in the context of a mid-afternoon snack. The observed, expected effect of the apple form on oral processing behavior is consistent with a previous report in adults (38). For the raw apple segment, the eating rate was 20 g/min here (apple segment) vs. 27 g/min in Forde et al.'s study (entire apple); for applesauce, it was 74 g/min in our study vs. 90 g/min in Forde et al.'s study. Children have slower eating rates (26 and 18% lower for the apple segment and puree, respectively) than adults, but the decrease in eating rate due to food form is comparable for both populations.

However, the apple form-induced satiation observed in adults was not observed in our study involving 8- to 10-year-old children. Although a proper dedicated study would be necessary to draw firm comparisons between adults and children, some methodological differences between Flood-Obbagy and Rolls' study (11) and ours can be considered to discuss these contrasting results. These are related to the study protocol, meal context, and properties of the test food.

First, concerning the study protocol, Flood-Obbagy and Rolls (11) fixed the preload ingestion duration. We did not, as we hypothesized that the oral exposure time was the main factor influencing intake and that fixing the ingestion rate may be a less natural and disturbing situation for children. The break duration after preload consumption and the subsequent food intake were slightly different in both studies (15 min from the start of the consumption of the preload vs. 10 min after the consumption of the preload in our study). As the preload ingestion rate was left to the discretion of the child, fixing the break duration made it possible for us to strictly control the duration between the end of the preload consumption and the *ad libitum* intake for all children eating more or less quickly and for both preloads. However, in both cases, the break durations were not long enough to induce post-ingestive effects (30, 31).

Second, the study in adults was realized during a lunch (11), whereas we evaluated this in the context of a mid-afternoon snack, a much simpler meal in terms of number of dishes and in terms of different symbolic values of foods socially considered appropriate for a meal or a snack. Children's eating behaviors might be somehow slightly different between lunch and mid-afternoon snacks. Indeed, a French sociological exploration reported that although the mid-afternoon snack is considered a meal, it resists nutritional injunctions and is associated with the universes of sweetness and pleasure (39).

Third, concerning the test foods, the ED of the *ad libitum* food used by Flood-Obbagy and Rolls (11) is much higher [2.2 kcal/g for the cheese tortellini and tomato sauce (64% of energy from carbohydrate, 16% energy from fat, and 20% of energy from protein)] than that of the one we chose (0.92 kcal/g for the sweetened cottage cheese); thus, the satiating properties of these two foods might be quite different. Another explanation to consider is that the sweetened cottage cheese might have been slightly too much liked by the children. This food was chosen because it was relatively neutral, but it was revealed to be as liked as the two preloads. This might have increased its consumption slightly beyond satiation, especially in an *ad libitum* situation that might contrast with usual home practice when parents decide the size of the portion to eat. During the study, children were clearly happy to serve themselves.

Finally, we chose an *ad libitum* food that requires very little oral processing while the previous authors chose a more textured food requiring chewing behavior (pasta). One can hypothesize that a similar phenomenon as for sensory-specific satiation but specific to the textural properties (which could be named “texture-specific satiation”) might explain why eating the apple segments first did not decrease the consumption of a cottage cheese, a food with different textural properties (and easier to eat from an oral processing perspective) than the food preload. Therefore, there may be a sensory control of the eating behavior for a succession of foods based on texture contrast. In this line, offering after a first food a second food with a different texture from the first one may reactivate the desire to eat. Not all mechanisms related to sensory-specific satiation have been understood so far. Nevertheless, sensory-specific satiation is known to be expressed differently in adults and children (40). Exploring this line of research is worthwhile to advance our understanding of the relation between texture perception (and oral processing capabilities) and appetite control abilities in children. Additionally, a better understanding of how children form expectations on satiating properties of a food based on texture is needed. In adults, as discussed by Nguyen and colleagues (41), it appears that “the effect of texture on satiety expectations is not a straightforward function of hard/soft or viscous/not viscous, but rather related to a number of factors: viscosity, food particles, the complexity of the food items, their interaction, and their influence on the temporality of the in-mouth perception.” Past experiences (i.e., familiarity) are also likely to be determinant (42): “children who ate the foods more often expected them to deliver greater satiation.” Thus, our study clearly calls for more research to understand the links between food oral processing and satiation in children. In this context, other studies considering the strategy of “concurrent evaluation” of intake (while controlling for differences in food acceptance and familiarity as discussed earlier) would be of interest to complement the present results obtained with the preload paradigm. Indeed, direct measurements of *ad libitum* intake of foods bearing textural differences would provide complementary information on the role of food texture on consumption in children.

Our study allowed us to explore the variability in eating rate for the different foods in the child population. We observed that *ad libitum* cottage cheese intake was highly correlated with its eating rate, validating previous results in children showing that fast eaters eat more during a meal (18). In addition, eating rates of cottage cheese and applesauce were correlated, whereas they were not correlated with those of the raw apple segments. Therefore, fast eaters of semisolid foods may not necessarily be fast eaters for chewable foods. In addition, eating rate and, more generally, eating behavior in children should be seen in light of the development of their chewing skills, which might impact the role of food texture properties in satiation processes. Dental status is particularly important to consider. A previous study suggested that dental caries may affect eating habits (21, 22). Another study conducted in a Finnish interventional study [Special Turku Coronary Risk Factor Intervention Project (STRIP)] suggested a link between dental maturity, BMI, and energy intake in 148 children aged 6 to 12 years (43). Overall, this calls for more work to describe developmental changes related to food oral abilities during this very specific time frame and its effects on children's eating behavior. However, eating fast is thought to be a modifiable phenotype implicated in the overweight problem, but the links with oral processing capacities have not been explored much so far.

In conclusion, this pioneering study calls for further research to better understand the interplay between the textural properties of food, oral processing behaviors, food intake, and appetite control abilities in children. It is necessary to highlight new levers to explore to foster healthy food intake in children, whether or not they have chewing difficulties.

## Data Availability Statement

The raw data supporting the conclusions of this article will be made available by the authors, without undue reservation.

## Ethics Statement

The studies involving human participants were reviewed and approved by Comité de Protection des Personnes Ile-de-France II N° IDRCB 2019-A00890-57. Written informed consent to participate in this study was provided by the participants' legal guardian/next of kin.

## Author Contributions

CS and CT designed the research with advice from SN. OP, CS, and CT conducted the research. ES performed the coding of all videotapes. CS and CT analyzed the data and wrote the paper. SN and OP critically revised the manuscript. All authors have primary responsibility for the content and approved the final draft.

## Conflict of Interest

The authors declare that the research was conducted in the absence of any commercial or financial relationships that could be construed as a potential conflict of interest.

## Abbreviations

BMI: body mass index
VAS: visual analog scale
ED: energy density
EI: energy intake
SR: satiety responsiveness
SE: slowness in eating
EF: enjoyment of food
FR: food responsiveness
FF: food fussiness
CEBQ: Child eating behavior questionnaire
ECC: early childhood caries.

## References

[1] WHO. World Health Organization (2020). Available online at: https://www.who.int/news-room/fact-sheets/detail/obesity-and-overweight (accessed March 03, 2021).

[2] Bolhuis DP Forde CG. Application of food texture to moderate oral processing behaviors and energy intake. Trend Food Sci Technol.(2020) 106:445–56. 10.1016/j.tifs.2020.10.021

[3] Teo PS Forde CG. The impact of eating rate on energy intake, body composition and health. In: Mayselman HL editor. Handbook of Eating and Drinking. Cham: Springer (2019). p. 1–27.

[4] Krop EM Hetherington MM Nekitsing C Miquel S Postelnicu L Sarkar A. Influence of oral processing on appetite and food intake—a systematic review and meta-analysis. Appetite. (2018) 1:253–69. 10.1016/j.appet.2018.01.018 29408331

[5] Stribitcaia E Evans CEL Gibbons C Blundell J Sarkar A. Food texture influences on satiety: systematic review and meta-analysis. Sci Rep. (2020) 10:12929. 10.1038/s41598-020-69504-y 32737349 PMC7395742

[6] de Graaf C. Texture and satiation: the role of oro-sensory exposure time. Physiol Behav. (2012) 107:496–501. 10.1016/j.physbeh.2012.05.008 22609070

[7] James B. Oral processing and texture perception influences satiation. Physiol Behav. (2018) 193(Pt B):238–41. 10.1016/j.physbeh.2018.03.015 29550537

[8] Larsen DS. Food texture, oral processing and satiation: examining their relationship. In: Melton L Shahidi F Varelis P editors. Encyclopedia of Food Chemistry. Oxford: Academic Press (2019). p. 150–3.

[9] Campbell CL Wagoner TB Foegeding EA. Designing foods for satiety: the roles of food structure and oral processing in satiation and satiety. Food Struct. (2017) 13:1–12. 10.1016/j.foostr.2016.08.002

[10] Robinson E Almiron-Roig E Rutters F de Graaf C Forde CG Tudur Smith C. A systematic review and meta-analysis examining the effect of eating rate on energy intake and hunger. Am J Clin Nutr. (2014) 100:123–51. 10.3945/ajcn.113.081745 24847856

[11] Flood-Obbagy JE Rolls BJ. The effect of fruit in different forms on energy intake and satiety at a meal. Appetite. (2009) 52:416–22. 10.1016/j.appet.2008.12.001 19110020 PMC2664987

[12] Goh J Russell C Liem D. An investigation of sensory specific satiety and food size when children consume a whole or diced vegetable. Foods. (2017) 6:55. 10.3390/foods6070055 28737712 PMC5532562

[13] Liem DG Russell CG. Supersize me. Serving carrots whole versus diced influences children's consumption. Food Qual Prefer. (2019) 74:30–7. 10.1016/j.foodqual.2019.01.006

[14] Bergamaschi V Olsen A Laureati M Zangenberg S Pagliarini E Bredie WLP. Variety in snack servings as determinant for acceptance in school children. Appetite. (2016) 96:628–35. 10.1016/j.appet.2015.08.010 26275333

[15] Werthmann J Jansen A Havermans R Nederkoorn C Kremers S Roefs A. Bits and pieces. Food texture influences food young children. Appetite. (2015) 84:181–7. 10.1016/j.appet.2014.09.025 25312750

[16] Laureati M Sandvik PL Almli V Sandell M Zeinstra GG Methven L. Individual differences in texture preferences among European children: development and validation of the child food texture preference questionnaire (CFTPQ). Food Qual Prefer. (2020) 80:103828. 10.1016/j.foodqual.2019.103828

[17] Fogel A Goh AT Fries LR Sadananthan SA Velan SS Michael N. A description of an 'obesogenic' eating style that promotes higher energy intake and is associated with greater adiposity in 4.5year-old children: results from the GUSTO cohort. Physiol Behav. (2017) 176:107–16. 10.1016/j.physbeh.2017.02.013 28213204 PMC5436621

[18] Fogel A Goh AT Fries LR Sadananthan SA Velan SS Michael N. Faster eating rates are associated with higher energy intakes during an ad libitum meal, higher BMI and greater adiposity among 4.5-year-old children: results from the Growing Up in Singapore Towards Healthy Outcomes (GUSTO) cohort. Br J Nutr.(2017) 117:1042–51. 10.1017/S0007114517000848 28462734 PMC5472197

[19] Berkowitz RI Moore RH Faith MS Stallings VA Kral TV Stunkard AJ. Identification of an obese eating style in 4-year-old children born at high and low risk for obesity. Obesity (Silver Spring). (2010) 18:505–12. 10.1038/oby.2009.299 19779474 PMC2917041

[20] Almotairy N Kumar A Trulsson M Grigoriadis A. Development of the jaw sensorimotor control and chewing—a systematic review. Physiol Behav. (2018) 194:456–65. 10.1016/j.physbeh.2018.06.037 29960013

[21] Linas N Peyron MA Hennequin M Eschevins C Nicolas E Delfosse C. Masticatory behavior for different solid foods in preschool children according to their oral state. J Texture Stud. (2019) 50:224–36. 10.1111/jtxs.12387 30636045

[22] Linas N Peyron MA Eschevins C Hennequin M Nicolas E Collado V. Natural food mastication capability in preschool children according to their oral condition: a preliminary study. J Texture Stud. (2020) 51:755–65. 10.1111/jtxs.12536 32442320

[23] Fogel A Fries LR McCrickerd K Goh AT Quah PL Chan MJ. Oral processing behaviours that promote children's energy intake are associated with parent-reported appetitive traits: results from the GUSTO cohort. Appetite. (2018) 126:8–15. 10.1016/j.appet.2018.03.011 29551400 PMC5973283

[24] Francou A Hébel P. Le goûter en perte de vitesse et loin des recommandations. in Consommations et modes de vie (N° 290, ISSN 0295-9976) CREDOC, Ed. 2017.

[25] Lange C Chabanet C Nicklaus S Visalli M Schwartz C. A dynamic method to measure the evolution of liking during food consumption in 8- to 10-year-old children. Food Qual Prefer. (2019) 71:510–16. 10.1016/j.foodqual.2018.07.012

[26] ANSES. Étude individuelle nationale des consommations alimentaires 3 (INCA 3). Avis de l'Anses, Rapport d'expertise collective. (2017). Available online at: https://www.anses.fr/en/system/files/NUT2014SA0234Ra.pdf (accessed March 03, 2021).

[27] Bouhlal S. Consequence of salt, sugar and fat content modifications in foods on children's preference and intake. (2011). Available online at: http://www.theses.fr/2011DIJOS110 (accessed October 10, 2021).

[28] Bouhlal S Issanchou S Nicklaus S. Mysterious fat. The different impact of fat content on toddlers' and adults' food intake. Appetite.(**2011)** 57:S6–S7. 10.1016/j.appet.2011.05.131

[29] de Saint Pol T Ricroch L. Le temps de l'alimentation en France. INSEE première N° 1417. (2012).

[30] Almiron-Roig E Palla L Guest K Ricchiuti C Vint N Jebb SA. Factors that determine energy compensation: a systematic review of preload studies. Nutr Rev. (2013) 71:458–73. 10.1111/nure.12048 23815144 PMC3746122

[31] Chapelot D. 2—Quantifying satiation and satiety. In: Blundell. JE Bellisle F editors. Satiation, Satiety and the Control of Food Intake. Cambridge: Woodhead Publishing (2013). p. 12–39.

[32] Schwartz C Lange C Hachefa C Cornil Y Nicklaus S Chandon P. Effects of snack portion size on anticipated and experienced hunger, eating enjoyment, and perceived healthiness among children. Int J Behav Nutr Phys Activity. (2020) 17:70. 10.1186/s12966-020-00974-z 32487121 PMC7268352

[33] Bakke M Bergendal B McAllister A Sjögreen L Asten P. Development and evaluation of a comprehensive screening for orofacial dysfunction. Swed Dent J. (2007) 31:75–84. 17695052

[34] Pena CR Pereira MM Bianchini EMG. Características do tipo de alimentação e da fala de crianças com e sem apinhamento dentário (Characteristics of food consistence and speech production in children with normal occlusion and malocclusion related to tooth crowding). Revista CEFAC.(2008) 10:58–67. 10.1590/S1516-18462008000100009

[35] Wardle J Guthrie CA Sanderson S Rapoport L. Development of the children's eating behaviour questionnaire. J Child Psychol Psychiatry Allied Disciplines. (2001) 42:963–70. 10.1111/1469-7610.00792 11693591

[36] de Onis M Onyango A Borghi E Siyam A Nishida C Siekmann J. Development of a WHO growth reference for school-aged children and adolescents. Bulletin of the World Health Organization. (2007) 85:661–8. 18026621 10.2471/BLT.07.043497 PMC2636412

[37] Kane L Wright C Fariza WF Hetherington M. Energy compensation in enterally fed children. Appetite. (2011) 56:205–9. 10.1016/j.appet.2010.11.002 21075153

[38] Forde CG Leong C Chia-Ming E McCrickerd K. Fast or slow-foods? Describing natural variations in oral processing characteristics across a wide range of Asian foods. Food Funct. (2017) 8:595–606. 10.1039/c6fo01286h 27883158

[39] Tibère L Rochedy A Sarrat C. Le goûter résiste à la nutritionnalisation. The “goûter” resists nutritionnalisation. Cahiers de Nutrition et de Diététique.(2018) 53:232–9. 10.1016/j.cnd.2018.03.008

[40] Olsen A Ritz C Hartvig DL Moller P. Comparison of sensory specific satiety and sensory specific desires to eat in children and adults. Appetite. (2011) 57:6–13. 10.1016/j.appet.2011.03.009 21477632

[41] Nguyen QC Wahlgren MB Almli VL Varela P. Understanding the role of dynamic texture perception in consumers' expectations of satiety and satiation. A case study on barley bread. Food Qual Prefer.(2017) 62:218–26. 10.1016/j.foodqual.2017.06.006

[42] Hardman CA McCrickerd K Brunstrom JM. Children's familiarity with snack foods changes expectations about fullness. Am J Clin Nutr.(2011) 94:1196–201. 10.3945/ajcn.111.016873 21918214

[43] Jääsaari P Tolvanen M Niinikoski H Karjalainen S. Advanced dental maturity of Finnish 6- to 12-yr-old children is associated with high energy intake. Eur J Oral Sci.(2016) 124:465–71. 10.1111/eos.12292 27644174

